# Supplementary material for: Content-rich biological network constructed by mining PubMed abstracts
Source: BMC Bioinformatics. 2004 Oct 8;5:147. doi: 10.1186/1471-2105-5-147 (PMC528731; doi:10.1186/1471-2105-5-147)
Supplement: Additional File 2 — The original results of the above study (non-essential files are deleted to keep the file size under the limit set by BMC bioinformatics). [file 1471-2105-5-147-S2.bz2 › chilibotAdditionalFile2/dip50/613ID10558980E10996/html/UBE3A_UBE2L3.html]

 


 **UBE3A** and **UBE2L3** 
  
Found 61 abstracts in PubMed, retrieved 50.  
 

 What does Google say? 
 PDF only 
| .edu only 

---

**Interactive relationship** (e.g. stimulation, inhibition, etc)

**Neutral relationship**- **UBE3A**  encodes  **UBE3A**  E6 AP  [ **UBE3A** ] , a ubiquitin protein ligase  [ **UBE2L3** ] , and shows brain specific imprinting, with brain expression predominantly from the maternal allele.  Ref: 11895368 Neurobiol Dis, 2002
- **UBE3A**  encodes a ubiquitin protein ligase  [ **UBE2L3** ]  and shows brain specific imprinting.  Ref: 9585605 Am J Hum Genet, 1998
- The E6 AP  [ **UBE3A** ]  gene  **UBE3A**  encodes an E3 ubiquitin protein ligase  [ **UBE2L3** ]  that binds the human papillomavirus E6 oncoprotein and catalyzes the ubiquitination of p53.  Ref: 9143503 Genomics, 1997
- The crystal structure of the E6AP  [ **UBE3A** ]  hect domain bound to the UbcH7  [ **UBE2L3** ]  ubiquitin conjugating enzyme E2 reveals the determinants of E2 E3 specificity and provides insights into the transfer of ubiquitin from the E2 to the E3.  Ref: 10558980 Science, 1999
- The region of E6AP  [ **UBE3A** ]  involved in complex formation with UbcH8 and UbcH7  [ **UBE2L3** ]  was mapped to its Hect domain.  Ref: 9153201 J Biol Chem, 1997
- AS is caused by maternal deletions for chromosome 15q11 q13, paternal uniparental disomy UPD, imprinting defects or loss of function mutations in the  **UBE3A**  locus which encodes E6 AP  [ **UBE3A** ]  ubiquitin protein ligase  [ **UBE2L3** ] .  Ref: 9887341 Hum Mol Genet, 1999
- also called ubiquitin protein ligase  [ **UBE2L3** ]  E3A  [ **UBE3A** ]  or  **UBE3A** , which functions as an ubiquitin protein ligase  [ **UBE2L3** ] .  Ref: 12619117 Hum Mutat, 2003
- Human papillomavirus HPV E6 protein forms a ternary complex with the cell cycle regulator p53 and the E6 associated protein E6AP  [ **UBE3A** ]  known as an E3 ubiquitin protein ligase  [ **UBE2L3** ] , leading to the degradation of p53 via the ubiquitination pathway.  Ref: 10974372 Antiviral Res, 2000
- Previously, it was shown that E6 AP  [ **UBE3A** ]  serves the role of a ubiquitin protein ligase  [ **UBE2L3** ]  E3 in the presence of the E6 protein from human papillomavirus types 16 and 18.  Ref: 9891052 Mol Cell Biol, 1999
- The  **UBE3A**  gene, which codes for the enzyme ubiquitin protein ligase  [ **UBE2L3** ]  involved in protein degradation and processing, has been found to be mutated in many but not all of patients with Angelman syndrome and can be considered a major Angelman candidate gene.  Ref: 10514831 Adv Neurol, 1999
- We have isolated a mouse cDNA clone containing the C2 domain of mouse Nedd4, a ubiquitin protein ligase  [ **UBE2L3** ]  E3 that also contains a hect homologous to the E6 AP  [ **UBE3A** ]  carboxyl terminus domain and three WW domains.  Ref: 10446181 J Biol Chem, 1999
- We demonstrate that UbcH7  [ **UBE2L3** ] , an E2 closely related to UbcH8, can also bind to E6AP  [ **UBE3A** ] .  Ref: 9153201 J Biol Chem, 1997
- The carboxyl terminal half of Rsp5 contains a HECT homologous to E6 AP  [ **UBE3A** ]  carboxyl terminus domain that catalytically ligates ubiquitin to proteins and functionally classifies Rsp5 as an E3 ubiquitin protein ligase  [ **UBE2L3** ] .  Ref: 10781604 J Biol Chem, 2000
- Our data show that the ubiquitin protein ligase  [ **UBE2L3** ]  function of E6 AP  [ **UBE3A** ]  is dispensable for its ability to coactivate nuclear hormone receptors, showing that E6 AP  [ **UBE3A** ]  possesses two separable independent functions, as both a coactivator and a ubiquitin protein ligase  [ **UBE2L3** ] .  Ref: 9891052 Mol Cell Biol, 1999
- Studies aimed at the identification of cellular targets for E6AP  [ **UBE3A** ] , an E3 ubiquitin protein ligase  [ **UBE2L3** ]  involved in ubquitin mediated degradation, led us to the identification of members of the Src family kinases as potential substrates for E6AP  [ **UBE3A** ] .  Ref: 10449731 Proc Natl Acad Sci U S A, 1999
- To correlate the E6 AP  [ **UBE3A** ]  coactivator function and ubiquitin protein ligase  [ **UBE2L3** ]  functions with the AS phenotype, we expressed mutant forms of E6 AP  [ **UBE3A** ]  isolated from AS patients and assessed the ability of each of these mutant proteins to coactivate PR or provide ubiquitin protein ligase  [ **UBE2L3** ]  activity.  Ref: 9891052 Mol Cell Biol, 1999
- The  **UBE3A**  gene encodes the E6 AP  [ **UBE3A** ]  ubiquitin protein ligase  [ **UBE2L3** ]  and has recently been shown to be mutated in Angelman syndrome patients who lack 15q11 q13 deletions or chromosome 15 paternal uniparental disomy.  Ref: 9465301 Genomics, 1998

**Non-interactive relationship** (e.g. studied together, co-existance, homology, etc.)

- Chromosomal mapping, sequencing and subcloning analyses indicated that the gene complementing uby1 1 is RSP5, which encodes a ubiquitin protein ligase  [ **UBE2L3** ]  E3 homologous to E6 AP  [ **UBE3A** ]  E6 associated protein.  Ref: 8752868 Genes Genet Syst, 1996
- Mutations in the Ubiquitin protein ligase  [ **UBE2L3** ]  3A gene  **UBE3A**  have been found in the latter group, but only preliminary figures are available on their frequencies.  Ref: 10737998 Hum Mutat, 2000
- The ubiquitin protein ligase  [ **UBE2L3** ]  E6 associated protein E6 AP  [ **UBE3A** ]  serves as its own substrate.  Ref: 9688277 Eur J Biochem, 1998
- 4 a mutation in the E3 ubiquitin protein ligase  [ **UBE2L3** ]  gene  **UBE3A** .  Ref: 11748306 J Med Genet, 2001
- Structure of an E6AP  [ **UBE3A** ]  UbcH7  [ **UBE2L3** ]  complex insights into ubiquitination by the E2 E3 enzyme cascade.  Ref: 10558980 Science, 1999
- Biochemical studies have shown that this occurs by targeted degradation of p53, dependent on the E6 AP  [ **UBE3A** ]  ubiquitin protein ligase  [ **UBE2L3** ] .  Ref: 9284524 Semin Cancer Biol, 1996
- Maternal truncation mutations in  **UBE3A** , which encodes for E6 AP  [ **UBE3A** ]  ubiquitin protein ligase  [ **UBE2L3** ] , have been shown to cause Angelman syndrome, which can also result from the absence of maternal chromosomal material from this region.  Ref: 10089011 Mol Psychiatry, 1999
- The hect domain protein family was originally identified by sequence similarity of its members to the C terminal region of E6 AP  [ **UBE3A** ] , an E3 ubiquitin protein ligase  [ **UBE2L3** ] .  Ref: 9575161 J Biol Chem, 1998
- VCY2 protein interacts with the HECT domain of ubiquitin protein ligase  [ **UBE2L3** ]  E3A  [ **UBE3A** ] .  Ref: 12207887 Biochem Biophys Res Commun, 2002
- This analysis revealed that in the majority of the AS patients examined, the ubiquitin protein ligase  [ **UBE2L3** ]  function of E6 AP  [ **UBE3A** ]  was defective whereas the coactivator function was intact.  Ref: 9891052 Mol Cell Biol, 1999
- The high risk human papillomavirus HPV E6 proteins stimulate the ubiquitination and degradation of p53, dependent on the E6AP  [ **UBE3A** ]  ubiquitin protein ligase  [ **UBE2L3** ] .  Ref: 11027293 Mol Cell Biol, 2000
- In the presence of E6 16, a third pair, E2 F1  [ **UBE2L3** ]  and E6 AP  [ **UBE3A** ]  mediate conjugation of myc by means of a mechanism that appears to be similar to that involved in the targeting of p53, formation of a myc.  Ref: 9653139 Proc Natl Acad Sci U S A, 1998
- Typical NEDD4 orthologues that contain a C2 Ca 2 lipid binding and a HECT Homologous to the E6 AP  [ **UBE3A** ]  Carboxyl Terminus ubiquitin protein ligase  [ **UBE2L3** ]  domain, and multiple WW domains have been shown to regulate the epithelial sodium channel ENaC.  Ref: 11840194 Eur J Hum Genet, 2001
- The cellular protein E6AP  [ **UBE3A** ]  functions as an E3 ubiquitin protein ligase  [ **UBE2L3** ]  in the E6 dependent ubiquitination of p53.  Ref: 9153201 J Biol Chem, 1997
- A candidate AS gene, ubiquitin protein ligase  [ **UBE2L3** ]  3A  **UBE3A**  E6 AP  [ **UBE3A** ] , has been identified, and mutations of this gene have been detected in several cases of AS.  Ref: 10732796 Neurogenetics, 1998
- In instances where there is no identifiable large deletion or UPD, the risk for recurrence may be as high as 50% as the result of either a maternally inherited imprinting center IC mutation or a ubiquitin protein ligase  [ **UBE2L3** ]   **UBE3A**  gene mutation.  Ref: 9557895 Am J Med Genet, 1998
- The E6 oncoprotein of human papillomaviruses HPVs that are associated with cervical cancer utilizes the cellular ubiquitin protein ligase  [ **UBE2L3** ]  E6 AP  [ **UBE3A** ]  to target the tumor suppressor p53 for degradation.  Ref: 11158620 Proc Natl Acad Sci U S A, 2001
- The E6 protein of the high risk human papillomaviruses HPVs and the cellular ubiquitin protein ligase  [ **UBE2L3** ]  E6AP  [ **UBE3A** ]  form a complex which causes the ubiquitination and degradation of p53.  Ref: 10864652 J Virol, 2000
- The ubiquitin protein ligase  [ **UBE2L3** ]  E6 associated protein E6 AP  [ **UBE3A** ]  was originally identified as a protein involved in the human papillomavirus E6 oncoprotein induced degradation of p53.  Ref: 9688277 Eur J Biochem, 1998
- Identification of members of the Src tyrosine kinase family as substrates of the E6AP  [ **UBE3A** ]  ubiquitin protein ligase  [ **UBE2L3** ]  implicates a role for the ubiquitin pathway in regulating the activities of individual members of this important family of signaling molecules.  Ref: 10449731 Proc Natl Acad Sci U S A, 1999
- ... Patient 4 had a mutation in the ubiquitin protein ligase  [ **UBE2L3** ]   **UBE3A**  saving the beta3 subunit gene.  Ref: 11198279 Ann Neurol, 2001
- Although the gene for E6 AP  [ **UBE3A** ]  ubiquitin protein ligase  [ **UBE2L3** ]   **UBE3A**  was mapped to the critical region for AS, evidence of expression from both parental alleles initially suggested that it was an unlikely candidate gene for this disorder.  Ref: 8988172 Nat Genet, 1997
- In addition, we also established that oxi 1, an oxidative stress responsive gene we previously cloned, encodes a family of proteins related to human E6 AP  [ **UBE3A** ]  ubiquitin protein ligase  [ **UBE2L3** ] .  Ref: 10408176 J Radiat Res Tokyo , 1999
- De novo truncating mutations in E6 AP  [ **UBE3A** ]  ubiquitin protein ligase  [ **UBE2L3** ]  gene  **UBE3A**  in Angelman syndrome.  Ref: 8988172 Nat Genet, 1997
- The E6 Ap  [ **UBE3A** ]  ubiquitin protein ligase  [ **UBE2L3** ]   **UBE3A**  gene is localized within a narrowed Angelman syndrome critical region.  Ref: 9110176 Genome Res, 1997
- One candidate is the ubiquitin protein ligase  [ **UBE2L3** ]  gene  **UBE3A** , which is maternally expressed only and localized to the 15q region.  Ref: 11489053 J Intellect Disabil Res, 2001
- We identified a number of positive interacting clones that encode ubiquitin protein ligase  [ **UBE2L3** ]  E3A  [ **UBE3A** ]   **UBE3A** .  Ref: 12207887 Biochem Biophys Res Commun, 2002
- It results from a maternal deletion of human chromosome 15q11 13 containing two candidate genes E6 P ubiquitin protein ligase  [ **UBE2L3** ]   **UBE3A**  and GABA A receptor beta3 subunit GABRB3, the latter of which has been also linked to autism.  Ref: 12672542 Neurosci Lett, 2003
- Human scribble Vartul is targeted for ubiquitin mediated degradation by the high risk papillomavirus E6 proteins and the E6AP  [ **UBE3A** ]  ubiquitin protein ligase  [ **UBE2L3** ] .  Ref: 11027293 Mol Cell Biol, 2000
- Imprinting mutations and loss of function mutations of E6 associated protein E6 AP  [ **UBE3A** ]  ubiquitin protein ligase  [ **UBE2L3** ]   **UBE3A** .  Ref: 9288101 Nat Genet, 1997
- Mapping data indicate that the entire transcriptional unit of the E6 AP  [ **UBE3A** ]  ubiquitin protein ligase  [ **UBE2L3** ]   **UBE3A**  gene lies within the AS region.  Ref: 9110176 Genome Res, 1997
- Candidate genes involved in the 15q region affected by duplication and deletion include the ubiquitin protein ligase  [ **UBE2L3** ]   **UBE3A**  gene responsible for Angelman syndrome and genes for three GABA A receptor subunits.  Ref: 9545097 Am J Med Genet, 1998
- Members of the hect domain protein family are characterized by sequence similarity of their C terminal regions to the C terminus of E6 AP  [ **UBE3A** ] , an E3 ubiquitin protein ligase  [ **UBE2L3** ] .  Ref: 10066826 J Biol Chem, 1999
- The human papilloma virus E6 associated protein E6AP  [ **UBE3A** ]  functions as a ubiquitin protein ligase  [ **UBE2L3** ]  E3 in the E6 mediated ubiquitination of p53.  Ref: 10373495 J Biol Chem, 1999
- On the basis of discoveries made in molecular biology, patients can be classified as belonging to five types deletion, paternal uniparental disomy UPD, imprinting defects, mutation of the  **UBE3A**  ubiquitin protein ligase  [ **UBE2L3** ]  gene and unidentified mechanism 15% 20% of patients.  Ref: 12373673 Rev Neurol, 2002
- Human papillomavirus type 16 E6 induces self ubiquitination of the E6AP  [ **UBE3A** ]  ubiquitin protein ligase  [ **UBE2L3** ] .  Ref: 10864652 J Virol, 2000
- Recently, several mutations have been found in the E6 AP  [ **UBE3A** ]  ubiquitin protein ligase  [ **UBE2L3** ]  gene  **UBE3A**  in a group of patients who are nondeleted and do not have uniparental disomy or imprinting defects.  Ref: 9600250 Hum Genet, 1998
- The E6AP  [ **UBE3A** ]  ubiquitin protein ligase  [ **UBE2L3** ]  E3 mediates the human papillomavirus induced degradation of the p53 tumor suppressor in cervical cancer and is mutated in Angelman syndrome, a neurological disorder.  Ref: 10558980 Science, 1999
- ... E6AP  [ **UBE3A** ]  accepted ubiquitins from Ubc4, UbcH5B, UbcH5C and UbcH7  [ **UBE2L3** ] .  Ref: 9990509 Genes Cells, 1998
- Point mutations were found in the gene for E6 AP  [ **UBE3A** ]  ubiquitin protein ligase  [ **UBE2L3** ]   **UBE3A**  identifying it as the AS gene, and tissue specific imprinting maternal expression was shown in the human brain and in hippocampal neurons and Purkinje cells in the mouse.  Ref: 9691003 Curr Opin Genet Dev, 1998
